# Plasmodium falciparum Malaria Presenting as a Thrombotic Thrombocytopenic Purpura (TTP) Mimic: A Case Report

**DOI:** 10.7759/cureus.56181

**Published:** 2024-03-14

**Authors:** Kalendra Kunwar, Sailesh Karki, Monika Jain, Sushma Edara, James Y Rixey, Frances Schmidt

**Affiliations:** 1 Internal Medicine, One Brooklyn Health/Interfaith Medical Center, Brooklyn, USA; 2 Internal Medicine, Brookdale University Hospital and Medical Center, Brooklyn, USA; 3 Pulmonary Medicine, Interfaith Medical Center, Brooklyn, USA

**Keywords:** adamts13, anemia, thrombocytopenia, ttp, falciparum malaria

## Abstract

Malaria can present with clinical manifestations overlapping with thrombotic thrombocytopenic purpura (TTP). We present the case of a 55-year-old female who presented with abdominal pain, fever, confusion, dehydration, and recent travel to Nigeria. Laboratory investigations were remarkable for low hemoglobin, decreased platelets, and elevated lactate. Suspicion for TTP occurred when the patient’s platelet count and hemoglobin progressively decreased along with acute kidney injury and confusion. There was an elevated ADAMTS13 antibody level and mildly reduced ADAMTS13 activity suggesting possible TTP. However, *Plasmodium falciparum* was seen on peripheral blood smears. Treatment with artemether-lumefantrine was initiated which led to improvement in parasitemia, platelet count, and anemia. The similarity between malaria and TTP is mostly explained by thrombotic microangiopathic anemia (TMA) present in both diseases. Awareness of the common pathogenesis of TMA in both diseases and clinical judgment are pivotal in determining the timely initiation of appropriate treatment.

## Introduction

Malaria is a life-threatening condition and remains a major killer in tropical countries. Malaria is caused by an infection of the parasite belonging to the genus *Plasmodium*. The most virulent is *Plasmodium falciparum*, causing one-third of the deaths associated with the disease [1]. Anemia in malaria is multifactorial [2], and quite often the presentation is that of microangiopathic hemolytic anemia with direct destruction of the red blood cells (RBCs) [3]. *Plasmodium falciparum* infection, responsible for severe malaria, leads to the adhesion of infected RBCs to blood vessel walls, causing endothelial cell activation, platelets, and white blood cell sequestration [4]. Thrombotic thrombocytopenic purpura (TTP) is caused by a deficiency of plasma metalloproteinase and ADAMTS13 and an increase in von Willebrand factor (VWF), leading to extensive microvascular thrombosis creating exceedingly high levels of shear stress and microangiopathic hemolysis [5]. Malaria and TTP are two serious medical conditions that can have similar symptoms, including fever, microangiopathic hemolytic anemia, thrombocytopenia, acute kidney injury, and changes in mental status [6,7]. This may potentially lead to misdiagnosis and delayed treatment. Similarities in the pathogenesis of thrombotic microangiopathic anemia (TMA) in both diseases have been reported previously in the literature. We present a case of *Plasmodium falciparum* malaria with microangiopathic hemolysis closely mimicking the presentation of TTP.

## Case presentation

A 55-year-old female presented to the emergency department (ED) with abdominal pain, loose stools, nausea, vomiting, and fever for 10 days. She had a recent travel history to Nigeria for four weeks and had returned to the United States two weeks ago. She did not receive any antimalarial prophylaxis during the travel. She had visited the ED a few days ago for similar complaints and was discharged on oral antibiotics. On presentation to the ED, she was ill-looking, pale, and dehydrated. On examination, the patient was confused and the mucous membranes were dry. The abdomen was soft and non-tender. Blood investigations showed low hemoglobin (10.6 mg/dL), low platelets (125,000/µL), lactate (2.3 mmol/L), prothrombin time/international normalized ratio (16.4/1.37), total bilirubin (2.2 mg/dL), lactate dehydrogenase (289 U/L), haptoglobin (<10 mg/dL), blood urea nitrogen/creatinine (35/1.7 mg/dL). Given her recent travel history, malaria, typhoid, and *Giardia *were the differential diagnoses. In addition, platelet counts and hemoglobin were progressively trending down (Table 1) raising the possibility of TTP.

**Table 1:** Laboratory investigations on different days of admission. MCV: mean corpuscular volume; MCH: mean corpuscular hemoglobin; LDH: lactate dehydrogenase; NA: not applicable

Parameters	Reference range and units	On admission	Day 2	Day 4	Day 7	Day 9
Hemoglobin	11.0–15.0 g/dL	10.6	8.5	6.8	6.0	9.2
Hematocrit	35–46%	32.1	25.1	21.5	18.0	28.4
MCV	80–100 fL	77.6	75.4	78.3	74.0	79.7
MCH	26.0–33.0 pg	25.8	25.5	24.7	24.8	25.9
Platelets	130–400 10^3^/µL	125	66	102	288	444
Retics	0.5–2%	0.57	0.59	0.37	2.58	7.78
LDH	140–271 U/L	287	289	291	419	393
Haptoglobin	17–317mg/dL	NA	<10	<10	<10	<10

**Figure 1:**
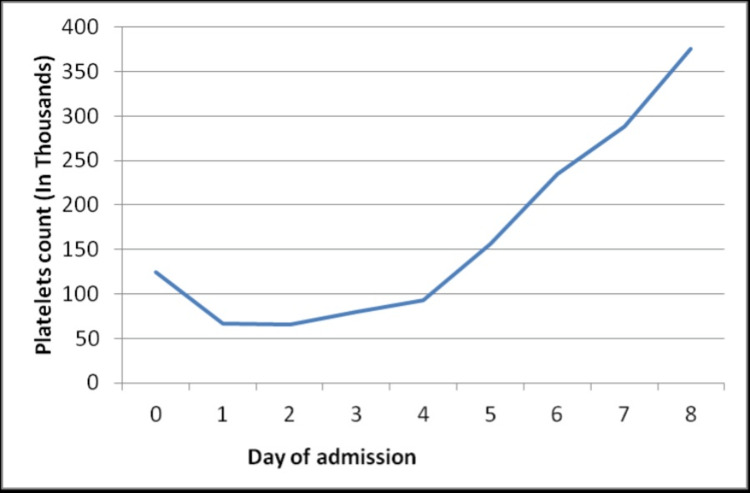
Platelet trend during the hospitalization.

**Figure 2:**
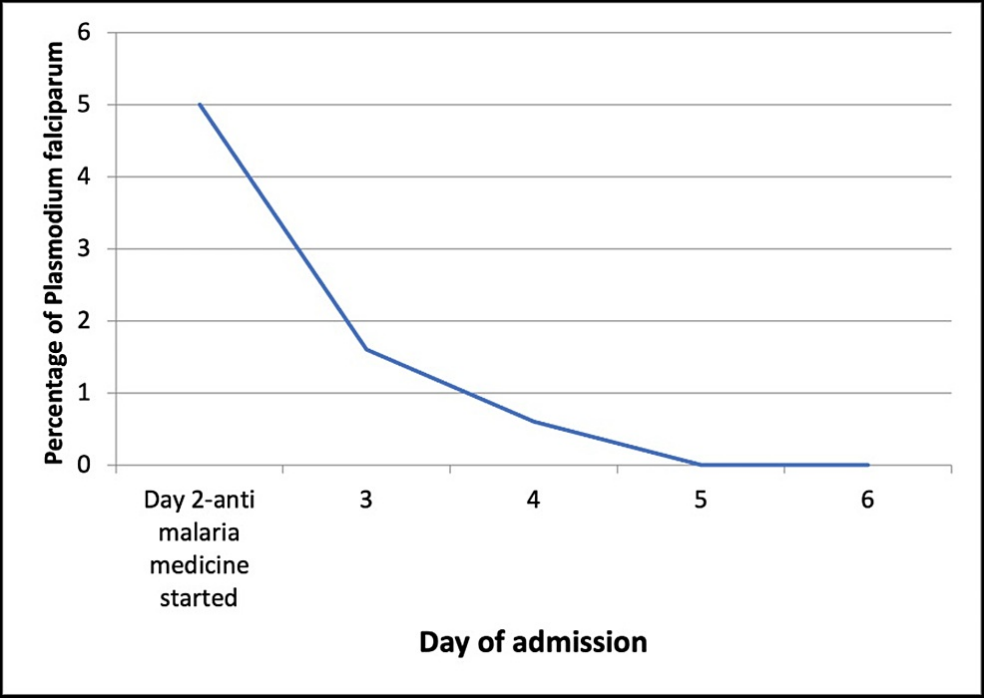
Downtrend of Plasmodium falciparum percentage with anti-malaria medicine.

Blood culture, stool for occult blood, stool culture, *Giardia *antigen, stool leukocytes, stool for clostridium difficile polymerase chain reaction, and toxins A and B were all negative. Chest X-ray and a CT scan of the abdomen and pelvis were negative. The PLASMIC score was 6 which was suggestive of a high probability of TTP. Thick and thin peripheral blood smear showed *Plasmodium falciparum* and occasional schistocytes. ADAMTS13 antibody was elevated at 22 U/mL (normal value: <12 U/mL) and reflex ADAMTS13 activity was 49.1 (normal value: >66.8; less than 10% relatively specific for TTP). She was managed with an artemether-lumefantrine combination for four days. With antimalarial medications, platelet counts gradually improved and normalized (Figure 1), and the percentage of RBCs infected with *Plasmodium falciparum* progressively decreased (Figure 2).

The patient remained afebrile after the second day of hospital admission. She reported gradual improvement in nausea, vomiting, and abdominal pain, with symptoms resolving on day five. She was able to tolerate food. The patient received two units of packed RBC transfusion during the hospital stay. After an improvement in her symptoms and normalization of her platelet count, the patient was discharged home. The patient denied any symptoms on follow-up after one month in the clinic and her laboratory investigations revealed normalization of hemoglobin (11.6 g/dL) and hematocrit (36.7%).

## Discussion

The similarity in clinical and laboratory presentation between malaria and TTP might be related to the presence of thrombotic microangiopathy in both diseases. Sinha et al. showed histological evidence of TMA in malaria in an observational study [8]. Similarities between the pathogenesis of thrombotic microangiopathy in malaria and TTP are evidenced by increased levels of VWF antibodies and decreased levels of ADAMTS13 antigen in severe malaria [9,10]. The reduction in ADAMTS13 activity might be caused by an unidentified inhibitor in malarial plasma [11]. The mildly attenuated ADAMTS13 activity in our patient might be related to a similar pathogenic mechanism.

TTP associated with malaria was initially reported by Nemie et al. in 2018. The patient was initially treated for *Plasmodium vivax* with artesunate and was managed with plasma exchange after TTP was suspected [12]. Subsequently, Ghadge et al. reported *Plasmodium falciparum* malaria associated with TTP in 2020. The patient had persistent altered mental status despite treatment for malaria and the mental status improved only after plasmapheresis [13]. However, in both cases, the diagnosis of TTP was made on clinical grounds after improvement with plasmapheresis, and ADAMTS13 testing was not done.

Similar to our case, Kurek et al. described a patient in 2023 who was initially suspected and treated for TTP. However, the confirmatory test for TTP was found to be negative, the patient was found to have malaria, and improvement was seen after initiation of intravenous artesunate [14]. This underscores the importance of a high index of suspicion of malaria, especially in patients with a travel history to endemic zones.

Positive antibodies to ADAMTS13 are a common finding in TTP as found in the UK TTP registry [15]. However, positive antibodies are found in many healthy individuals as well and are not specific to TTP. False-positive antibodies are more common in patients with systemic lupus erythematosus and antiphospholipid syndrome [16]. ADAMTS13 activity levels of less than 10% are used to confirm the diagnosis in appropriate clinical settings [17,18]. In our patient, ADAMTS13 activity was greater than 10% despite positive ADAMTS13 antibody levels which made TTP unlikely. The clinical presentation in our case was compatible with malaria as well as with TTP; however, our patient did not undergo plasmapheresis and she responded well to antimalarial medications.

## Conclusions

The striking clinical and laboratory resemblances between malaria and TTP with the presence of TMA suggest a possibility of shared pathogenic mechanisms involving VWF and ADAMTS13 in both diseases. ADAMTS13 should be monitored, especially in severe malaria cases with TMA. Decreased ADAMTS13 activity in combination with increased VWF may play a role in the complications of malaria. It is unknown if patients with malaria should be treated for TTP only if ADAMTS13 activity level is less than 10% or if there is no clinical improvement despite antimalarial treatment and resolved parasitemia when ADAMTS13 is lower than normal. ADAMTS13 in malaria might be the result of an unidentified inhibitor and the pathogenesis of TMA may be identical in both diseases. Awareness of the association of malaria with TTP may lead to early initiation of lifesaving treatment in appropriate patients. Clinical judgment and ADAMTS13 activity level remain the cornerstone in deciding on the need for plasmapheresis.
